# Supplementary material for: Dynamical control enables the formation of demixed biomolecular condensates
Source: Nat Commun. 2023 Nov 24;14:7678. doi: 10.1038/s41467-023-43489-4 (PMC10667521; doi:10.1038/s41467-023-43489-4)
Supplement: Supplementary file 1 — Supplementary information [file 41467_2023_43489_MOESM1_ESM.pdf]

Supplementary Information for:

Dynamical control enables the formation of demixed biomolecular condensates

Andrew Z. Lin [a,1], Kiersten M. Ruff [b,1], Furqan Dar [b,1], Ameya Jalihal [c], Matthew R. King [b], Jared M. Lalmansingh [b], Ammon E. Posey [b], Nadia A. Erkamp [b,d], Ian Seim [c], Amy S. Gladfelter [c,✉], Rohit V. Pappu[a,✉]

[a] Division of Biology and Biomedical Sciences, Plant and Microbial Biosciences, Washington University in St. Louis, St. Louis, MO 63130, USA

[b] Department of Biomedical Engineering and Center for Biomolecular Condensates, James F. McKelvey School of Engineering, Washington University in St. Louis, St. Louis, MO 63130, USA

[c] Department of Cell Biology, Duke University, Durham, NC 27708, USA

[d] *Yusuf Hamied Department of Chemistry, Centre for Misfolding Diseases, University of Cambridge, Lensfield Road, Cambridge, CB2 1EW, UK*

[1]These authors contributed equally

✉e-mail: amy.gladfelter@duke.edu; pappu@wustl.edu;

Supplementary Table 1: Frequencies of Monte Carlo moves for three-component mixture simulations

Move Type	Normalized By Min	Frequency
Rotation	500	4.75
Local	5000	47.52
Co-Local	1000	9.50
Multi-Local	500	4.75
Chain Reptation	500	4.75
Chain Translation	1000	9.50
Aniso. Cluster Translation (Small)	10	0.10
Aniso. Cluster Translation (Large)	1	0.01
Chain Pivot	1000	9.50
Double Pivot	1000	9.50
Cluster Translation (Small)	10	0.10
Cluster Translation (Large)	1	0.01

Details about the different moves can be found in the original LaSSI work, Choi et. al. [1], and the Supporting Information Appendix of Kar et. al. [2].

Supplementary Table 2: Numbers of molecules and box-sizes for two-component phase diagrams

Molecule 1	Molecule 2	L_1	L_2	L_3	L_4	L_5
185	4815	46	55	67	81	99
256	4744	51	61	74	90	109
442	4558	61	73	89	108	131
638	4362	69	83	101	122	148
834	4166	76	92	111	135	163
1030	3970	81	98	118	144	174
1303	3697	88	106	129	156	189
1618	3382	95	115	139	168	204
1814	3186	98	118	143	174	211
2206	2794	105	127	154	186	226
2500	2500	109	132	159	193	234
2794	2206	114	138	167	202	245
3186	1814	119	144	174	211	256
3382	1618	121	146	177	215	260
3697	1303	125	151	183	222	269
3970	1030	128	155	187	227	275
4166	834	130	157	190	231	280
4362	638	132	159	193	234	284
4558	442	134	162	196	238	288
4744	256	136	164	199	241	293
4904	96	137	165	201	243	295
4950	50	138	167	202	245	297

Supplementary Table 3: MC move frequencies for two-component phase diagrams

Move Type	Normalized by Min	Frequency
Rotation	500	14.24
Local	1000	28.48
Co-Local	666	18.99
Multi-Local	500	14.24
Chain Reptation	333	9.49
Chain Pivot	166	4.75
Double Pivot	166	4.75
Chain Translation	166	4.75
Anisotropic Cluster Translation (Small)	10	0.28
Anisotropic Cluster Translation (Large)	1	0.03

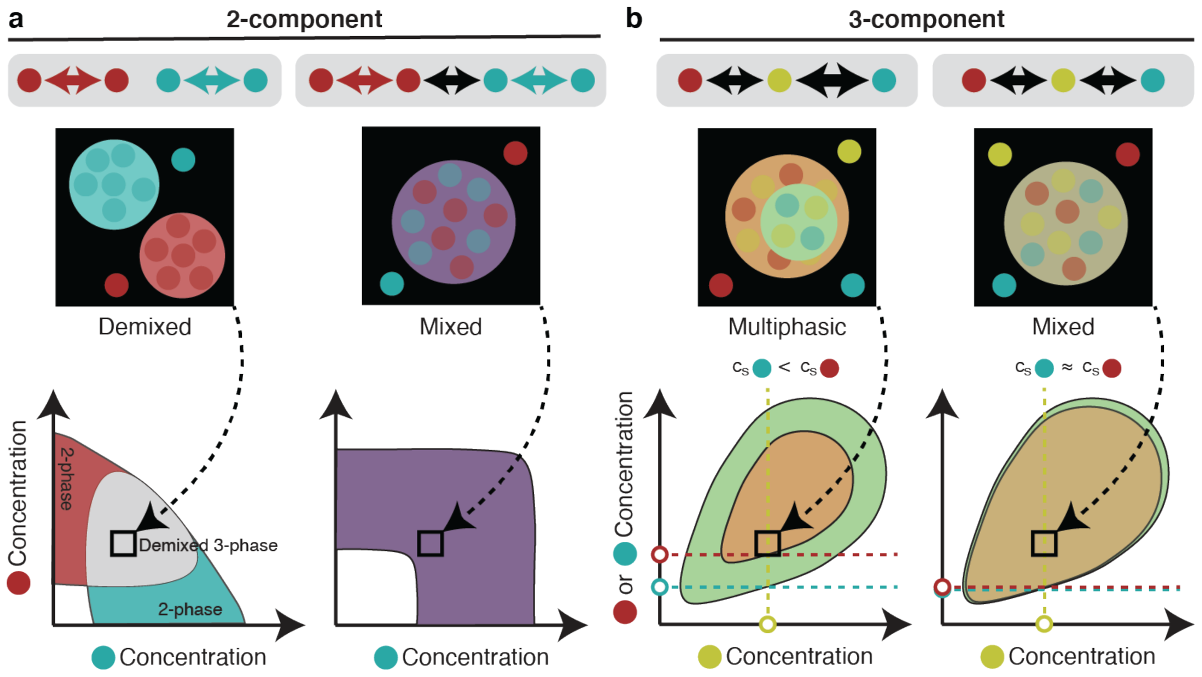

Supplementary Fig. 1: The interplay between homotypic and heterotypic interactions dictates the thermodynamic preferences for formed different types of condensates in binary ternary mixtures of macromolecules. (a) Left: Example phase behavior for a two-component system in which only homotypic interactions are present. Non-grey color regimes of the phase diagram denote a single condensate type is formed at these conditions, whereas the grey region denotes the region where demixed condensates can form. Right: Example phase behavior of a two-component system in which homotypic and heterotypic interactions are equivalent. Black arrows denote heterotypic interactions, whereas colored arrows denote homotypic interactions. (b) Example phase behavior of a three-component system in which the interaction strength of the yellow component with the two other components is asymmetric (left) or symmetric (right). Green area is the two-phase regime of the two-component system of the yellow and blue molecules, whereas the brown-orange area is the two-phase regime of the two-component system of yellow and red molecules. Here, c_s denotes the saturation concentration at the given starting concentration of the yellow molecule. Drawn to summarize the results of Lu et al., [3].

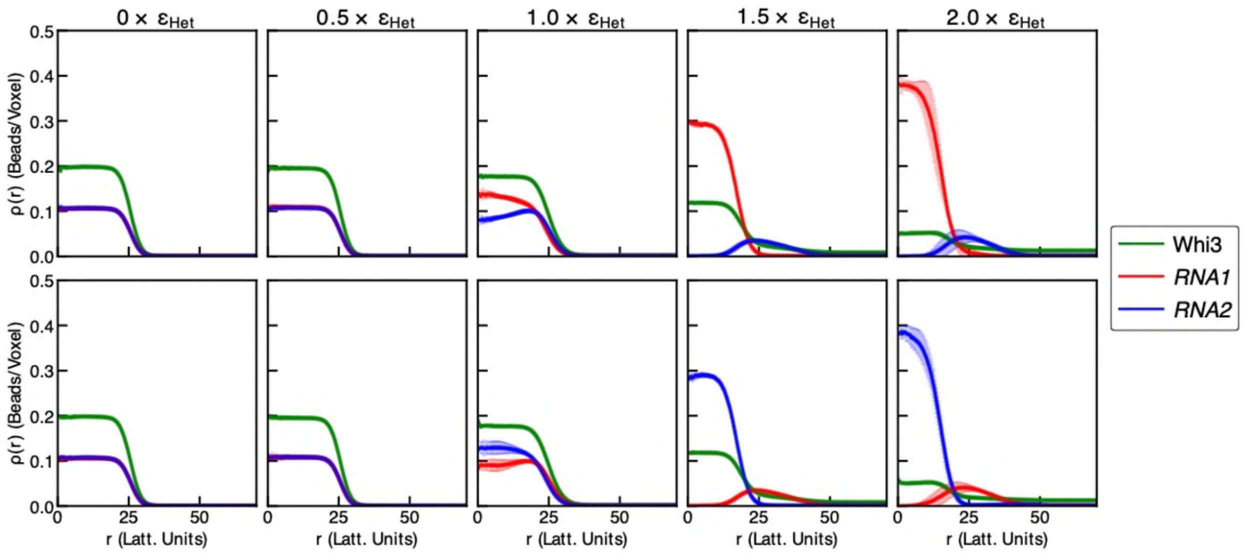

Supplementary Fig. 2: Density profiles from LaSSI lattice-based simulations of a *RNA1* (red), *RNA2* (blue), and Whi3 (green) system in which the strength of homotypic interactions of *RNA1* and *RNA2* are titrated with respect to a fixed strength of Whi3-*RNA* interactions. Here, ε_{Het} = -2k_BT and refers to the interaction strength between Whi3-*RNA1* and Whi3-*RNA2*. Each column summarizes results obtained by titrating homotypic *RNA1*-*RNA1* and *RNA2*-*RNA2* interaction strengths as a function of ε_{Het}. The top and bottom rows show the density profiles using the centers-of-mass of *RNA1* and *RNA2*, respectively. Three independent replicas were performed per condition and error bars denote standard errors of mean. Source data are provided as a Source Data file.

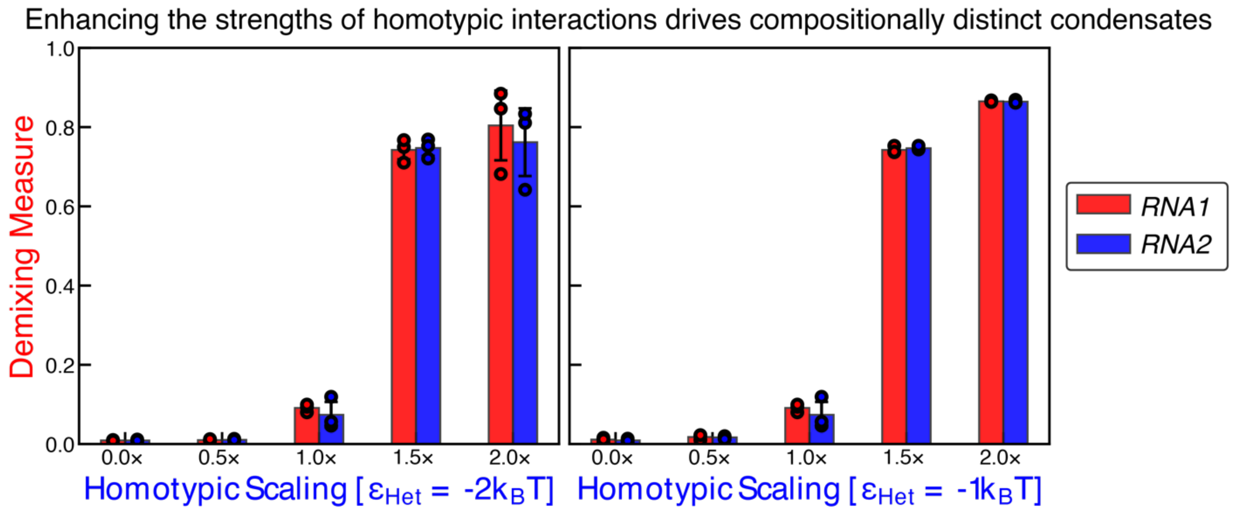

Supplementary Fig. 3: Demixing measure (see methods) as a function of homotypic interactions of *RNA1* and *RNA2* scaled by the heterotypic interaction strength, ε_{Het}. Here, circles denote three independent replicas. Error bars denote the standard error of mean. Source data are provided as a Source Data file.

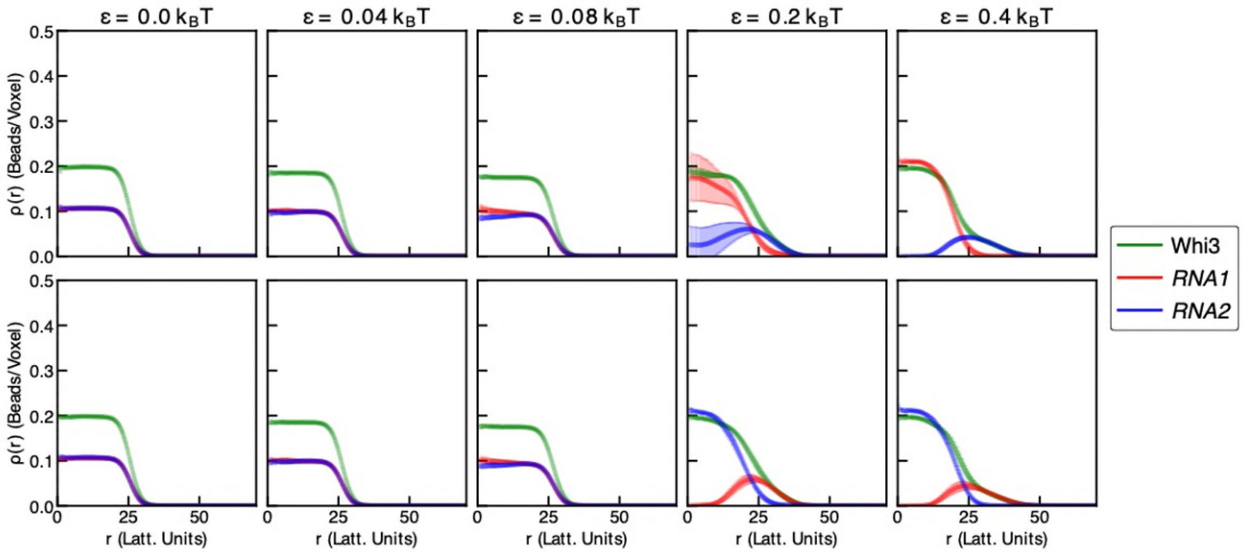

Supplementary Fig. 4: Radial density profiles from LaSSI lattice-based simulations of a *RNA1* (red), *RNA2* (blue), and Whi3 (green) system in which the strengths of repulsions between *RNA1* and *RNA2* are titrated. Here, each column denotes the heterotypic *RNA1*-*RNA2* repulsion strength denoted by ε. The Whi3-*RNA* heterotypic interaction strength is set to $\varepsilon_{\text{Het}} = -2k_BT$. The top and bottom rows show the density profiles from the centers-of-mass *RNA1* and *RNA2*, respectively. Three independent replicas were performed per condition and error bars denote standard errors of mean. Source data are provided as a Source Data file.

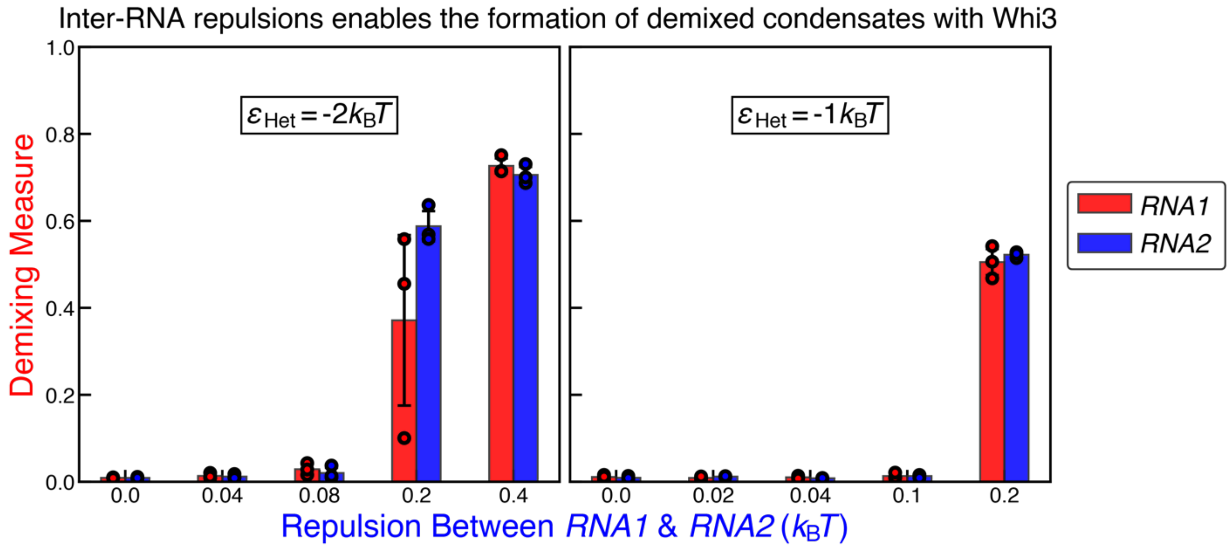

Supplementary Fig. 5: Demixing measure as a function of the repulsion strength between *RNA1* and *RNA2*. Here, circles denote three independent replicas. Error bars denote the standard error of mean. The data show that demixing increases as the strengths of the heterotypic inter-RNA repulsions increases. Source data are provided as a Source Data file.

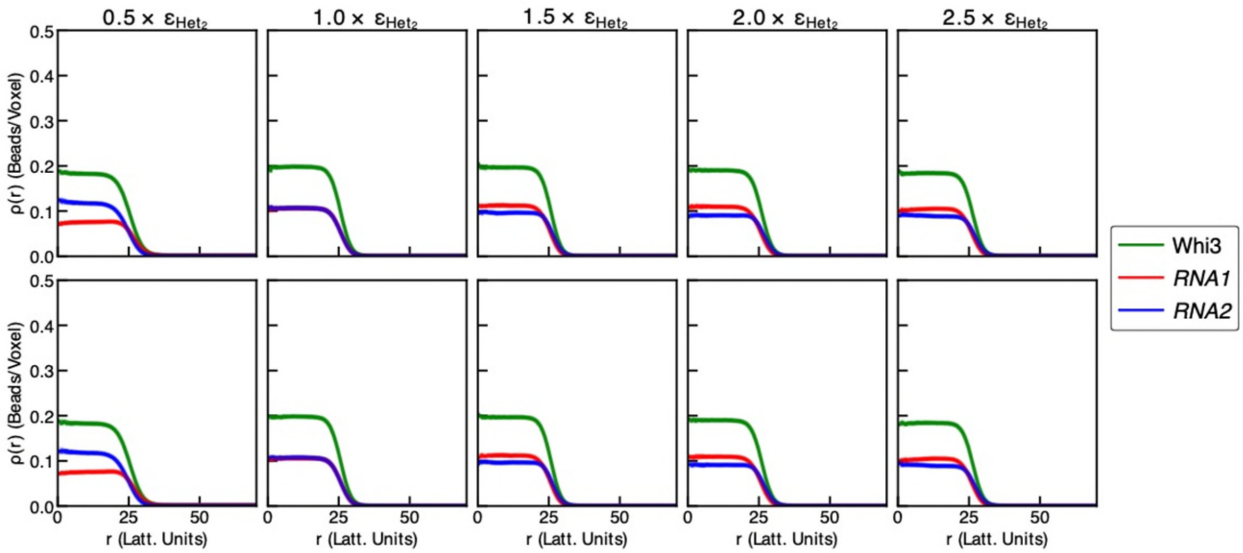

Supplementary Fig. 6: Radial density profiles from LaSSI lattice-based simulations of a *RNA1* (red), *RNA2* (blue), and Whi3 (green) system in which the strength of the Whi3-*RNA1* interaction is titrated with respect to the Whi3-*RNA2* interaction strength. Here, $\varepsilon_{Het2} = -2k_BT$ and refers to the interaction strength between Whi3-*RNA2*. Each column denotes the Whi3-*RNA1* interaction strength as a function of ε_{Het2}. The top and bottom rows show the density profiles from the centers-of-mass of *RNA1* and *RNA2*, respectively. Three independent replicas were performed per condition and error bars denote standard errors of mean. Source data are provided as a Source Data file.

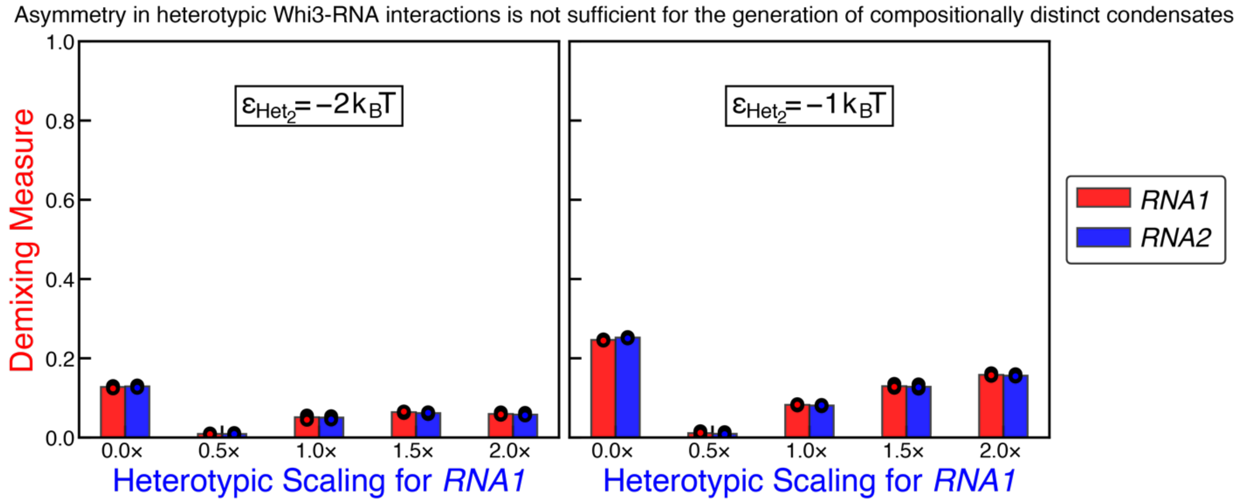

Supplementary Fig. 7: Demixing measure as a function the heterotypic interaction strength of Whi3-*RNA1*. Here, circles denote three independent replicas, ε_{Het2} refers to the interaction strength between Whi3-*RNA2*, and the Whi3-*RNA1* interaction strength is defined as a function of ε_{Het2}. Error bars denote the standard error of mean. Source data are provided as a Source Data file.

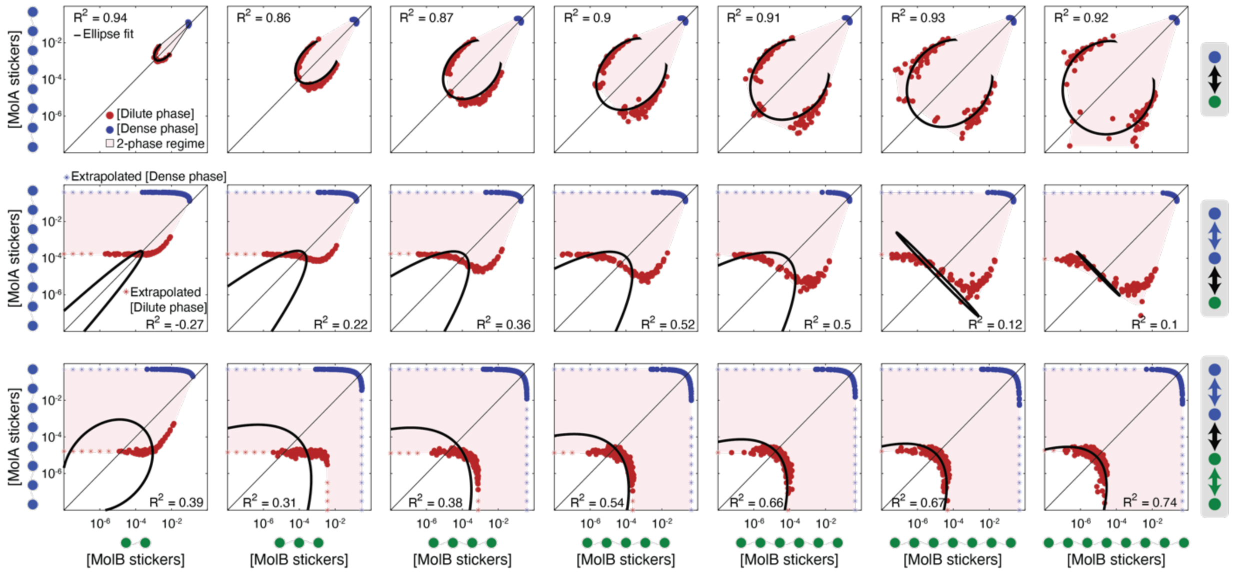

Supplementary Fig. 8: Phase diagrams from LaSSI simulations of two-component systems. Here, the valence of molecule A is always set to eight stickers and the valence of molecule B is titrated from two to eight. Concentrations are given in stickers / voxel. The top row shows data for a system where only heterotypic interactions between A and B molecules are present. The middle row shows data for a system where the homotypic interactions between A molecules are equivalent to the heterotypic interactions with molecule B, although homotypic interactions among B molecules are absent. The last row shows data for a system where homotypic interactions between molecules of type A and B are present and the strengths of these interactions are equivalent to heterotypic interactions between A and B molecules. Note that for this system, when molecule B has a valence of two, molecule B cannot phase separate on its own and thus the two-phase regime does not extend to the x-axis. Extrapolations of points along the phase boundary are determined by finding the minimum concentration of the dilute or dense phase boundary in which phase separation occurs for all systems that show a flattened boundary as the concentration of the other molecule decreases. Two independent replicas were performed per condition. Source data are provided as a Source Data file.

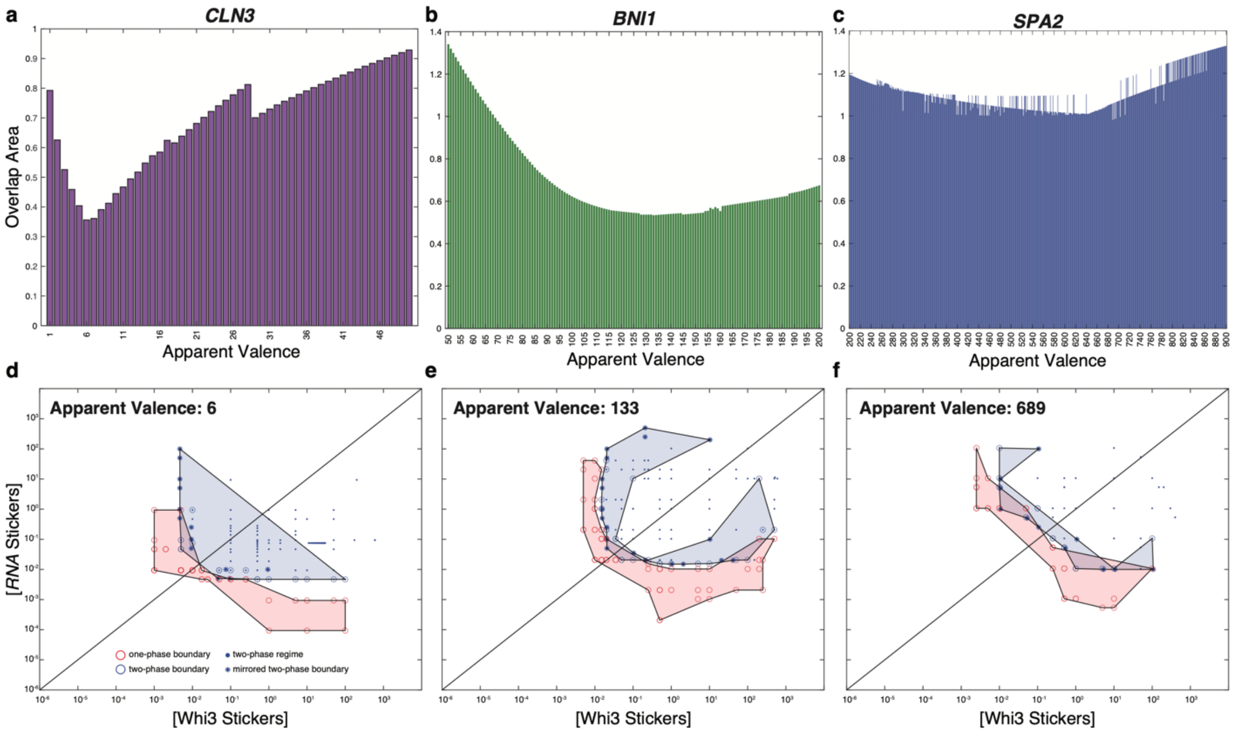

Supplementary Fig. 9: Dilute and dense phase overlap titration analysis. (a-c) Overlap area of the one-phase and two-phase regimes given the apparent valence of the *CLN3*, *BNI1*, and *SPA2*, respectively (see Methods). (d-f) Plot of the one-phase and two-phase boundary regimes for the apparent valence with the lowest overlap area. One-phase regimes and the two-phase boundary areas are shown in red and blue, respectively. Source data are provided as a Source Data file.

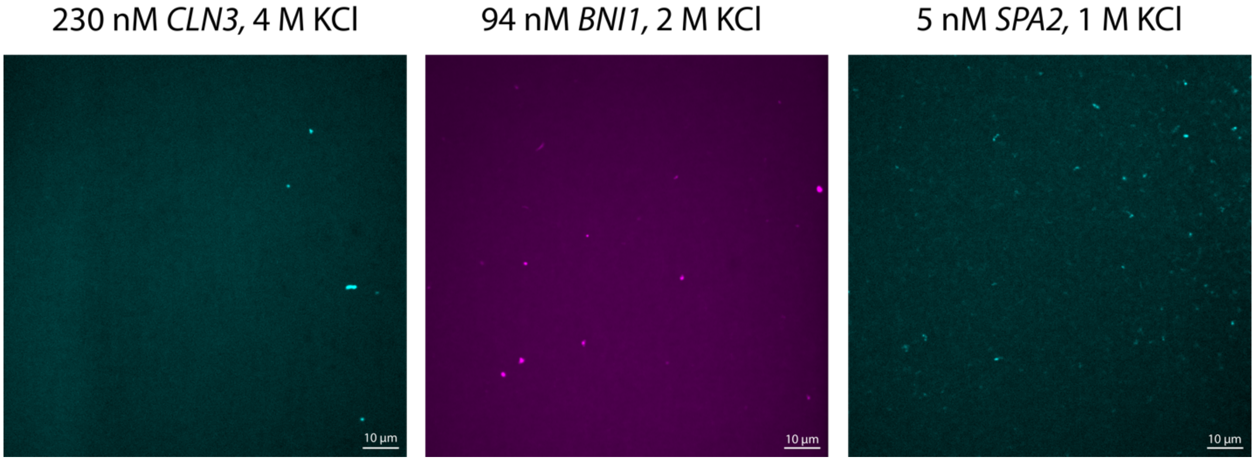

Supplementary Fig. 10: RNA-only condensates form, albeit weakly, at concentrations of RNA and monovalent salt that are distinctly different from the conditions used to generate Whi3-RNA condensates. The RNA and salt concentrations for each of the constructs are shown at the top of each panel. Samples in the absence of Whi3 were incubated at room temperature on a glass coverslip for 30 minutes before imaging. RNA stocks in water were diluted with 4.5 M KCl to achieve final concentrations of KCl listed.

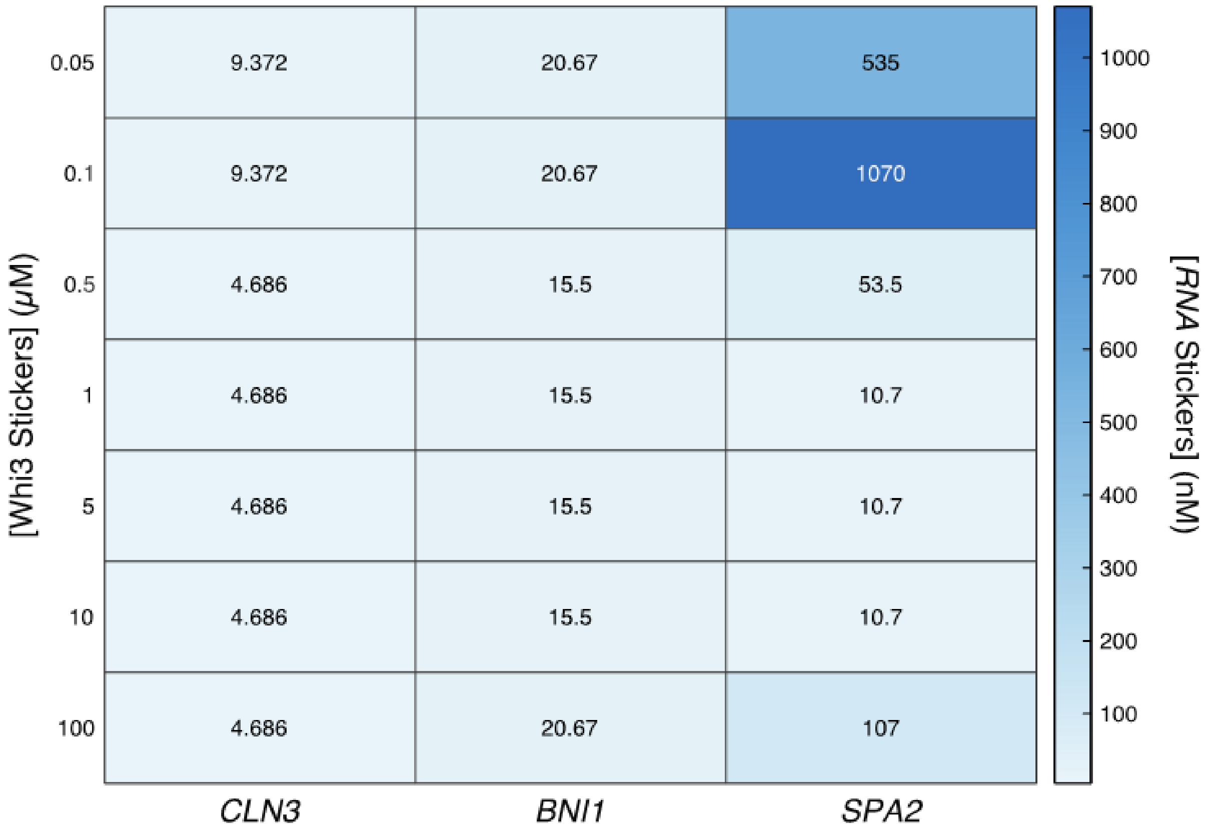

Supplementary Fig. 11: Quantitative summary of the concentrations of Whi3 and RNA stickers along the dilute arms of phase boundaries that define the condensates for Whi3 with *CLN3*, *BNI1*, and *SPA2*. The concentrations of Whi3 stickers are annotated as labels along the ordinate. The different columns correspond to different RNA molecules. Within each cell, the concentration of RNA stickers that defines the phase boundary via heterotypic interactions with Whi3 is marked numerically within the cell and color-coded using the color bar shown on the right. For example, for a concentration of 0.05 µM of Whi3 stickers, the concentration of RNA stickers that corresponds to the phase boundary in binary Whi3-RNA mixtures is ~ 9.4 nM, ~ 20.7 nM, and ~535 nM for *CLN3*, *BNI1*, and *SPA2*, respectively. As the concentration of Whi3 stickers increases, the concentrations of RNA stickers required to drive phase separation decreases. The converse is also true, and these results reinforce the importance of heterotypic interactions as drivers of condensation in Whi3-RNA mixtures.

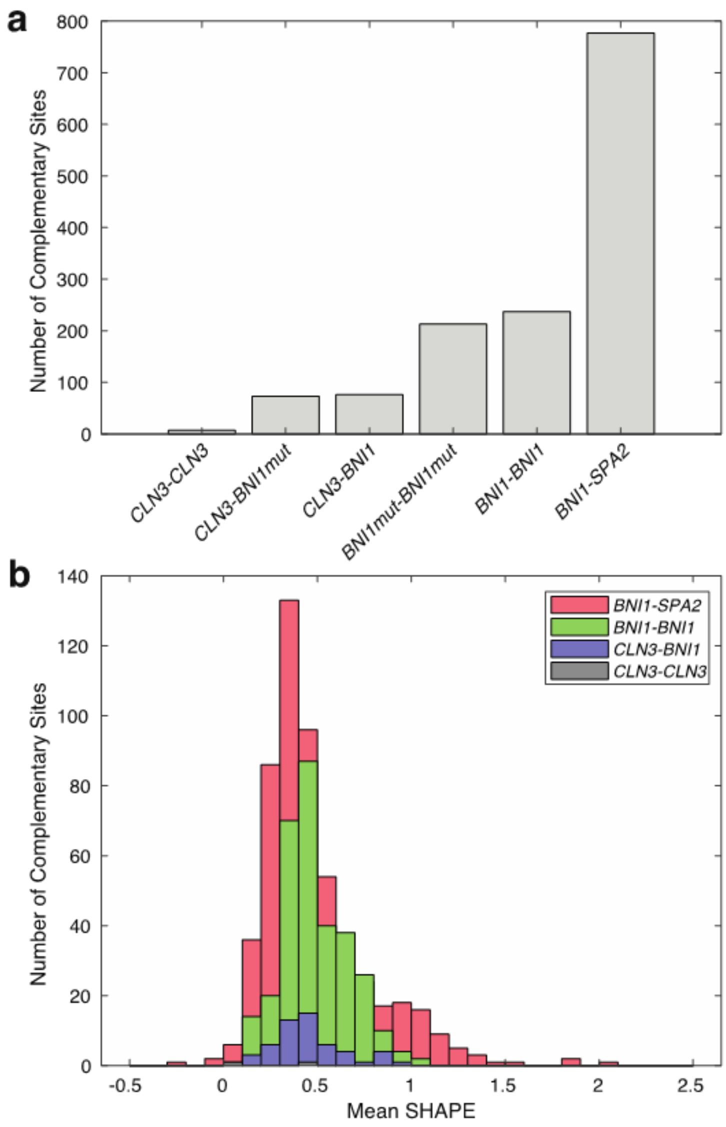

Supplementary Fig. 12: Number and accessibility of complementary sites between pairs of RNAs. (a) Number of GUUGle identified complementary sites for different pairs of RNA molecules [4]. (b) Histogram of mean values from the SHAPE-MaP analysis [5-7] for all nucleotides in both complementary sites. Larger SHAPE values imply those complementary sites are more accessible for intermolecular interactions. Source data are provided as a Source Data file.

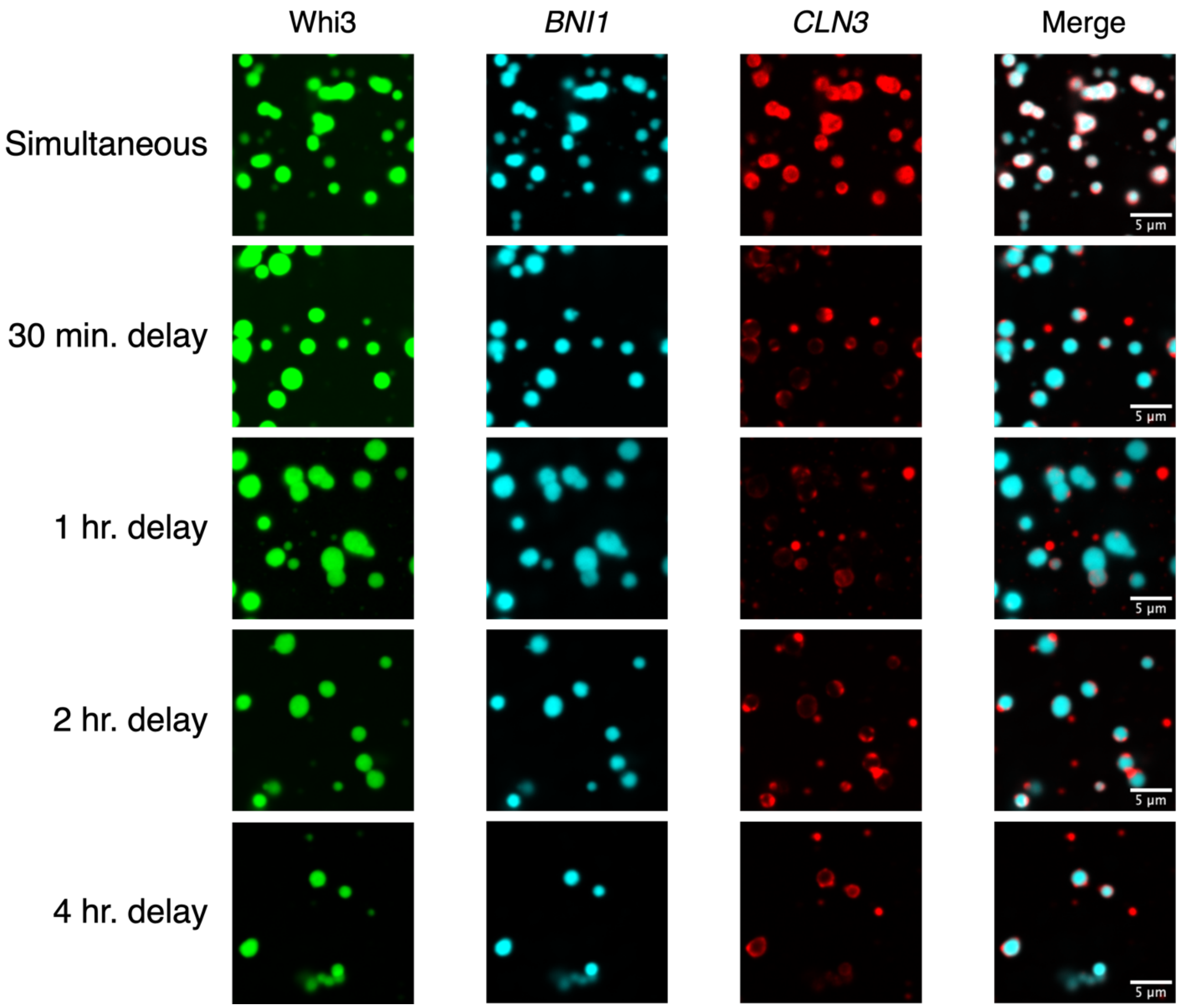

Supplementary Fig. 13: Analysis of how titration of the delay in adding the second RNA influences the degree of colocalization. Here, condensates were formed using Whi3 and *BNI1*. The *CLN3* molecules were added after a delay of 30 min, 1 hr, 2 hrs, or 4 hrs. The top row shows condensates formed using the *simultaneous* mode. Unlike the results shown in the top row, condensates formed in the *delayed* mode show a lack of colocalization of *BNI1* and *CLN3*. Therefore, well-mixed condensates form when all components are simultaneously added. Conversely, compositionally distinct Whi3-RNA condensates, with Whi3 being the shared component, form even with only a 30-minute delay in the addition of *CLN3* to pre-existing Whi3-*BNI1* condensates.

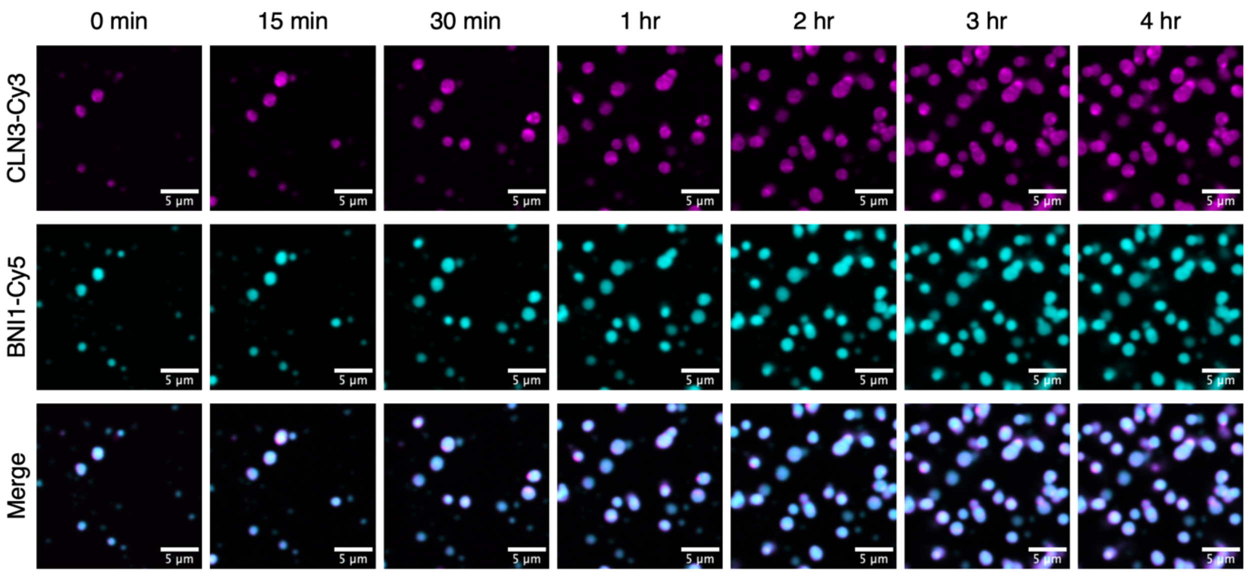

Supplementary Fig. 14: Time-lapse analysis of the colocalization of condensates that were prepared in the *simultaneous mode*. The condensates are formed from ternary mixtures of Whi3, *BNI1*, and *CLN3*. The Whi3 molecules were unlabeled, whereas *BNI1* is labeled with Cy5, and *CLN3* is labeled with a Cy3 tag. Images were collected at intervals of 15 min, 30 min, 1 hr, 2 hrs, 3 hrs, and 4 hrs after the initial simultaneous addition of all three components. The fields of view remain the same for all images. For all time points studied, the Pearson's r, which quantifies the degree of colocalization, is between 0.9 and 0.96. We propose that the data shown here are suggestive of the well-mixed condensates being thermodynamic ground states. This proposal is based on two other observations, including the results of Langdon et al., [7] who showed that preparing condensates following heat treatments of the RNA molecules of interest, leads to well-mixed condensates as opposed to demixed condensates. Similar results were reported by Boeynaems et al.,[8] in their study of ternary mixtures of arginine-rich peptides and different, base-pairing RNA molecules. Heating and annealing assays will drive unfolding of both the RNA and Whi3 RRM. The annealing protocol would have to be sufficiently slow to allow for refolding of the molecules and remodeling of the condensates. An optimal protocol for achieving this remains elusive. Therefore, for now, we propose, based on precedents in the literature, that well-mixed condensates are likely to be the thermodynamic ground states. A corollary of this proposal is that demixed, compositionally distinct condensates with Whi3 as the shared component are metastable.

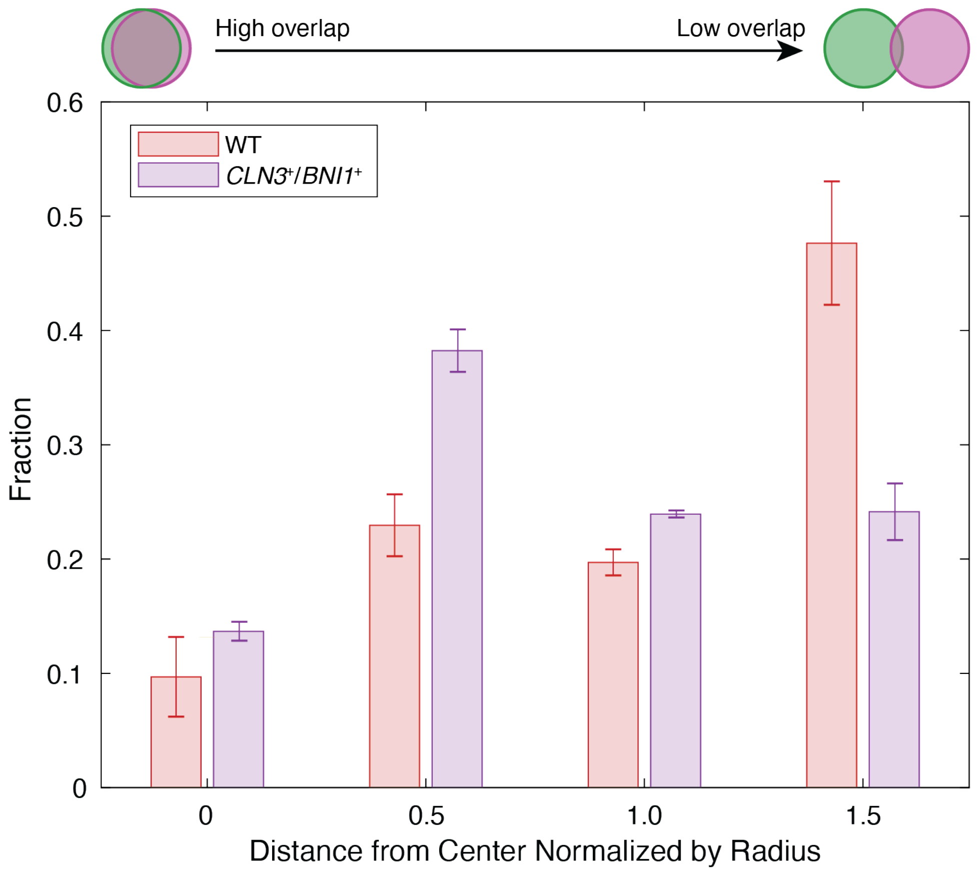

Supplementary Fig. 15: Histogram of distance from center normalized by radius for all *CLN3* and *BNI1* spots within 2 radii from each other. Here, the bin size was set to 0.5. Error bars denote the standard error across three independent experiments. Source data are provided as a Source Data file.

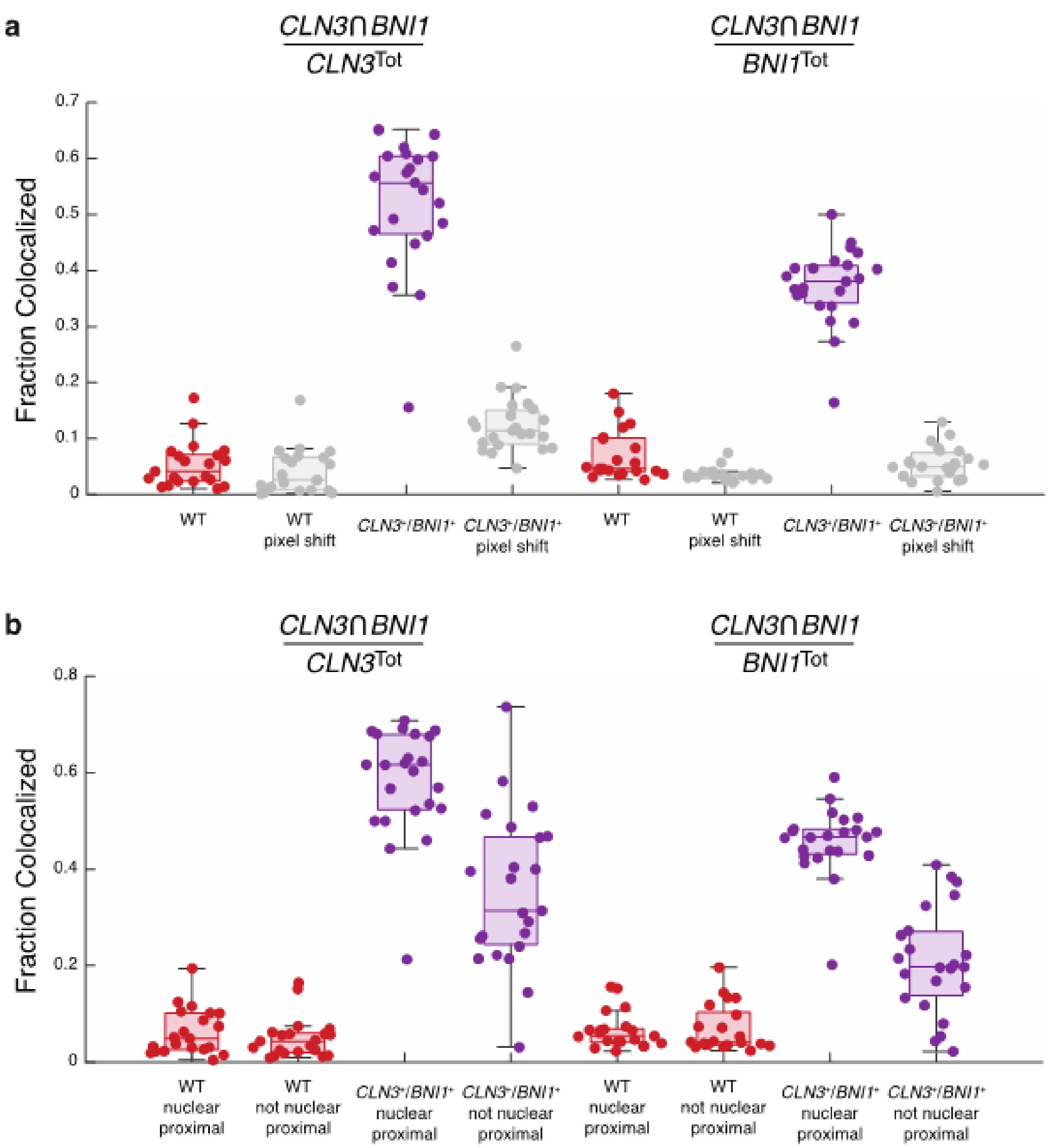

Supplementary Fig. 16: Pixel shift and subcellular localization analysis of *CLN3* and *BNI1* colocalization. (a) Fraction colocalized in wildtype (WT) and $CLN3^{+}/BNI1^{+}$ cells with pixel shift data. Here, either *CLN3* or *BNI1* identified spots were shifted by $2r$ in both the x and y direction, where r is the radius of the spot (see Methods). (b) Fraction colocalized based on nuclear proximity. Spots within $r+R$ away from the center of any nucleus were defined to be nuclear proximal, where R is the radius of the nucleus (see Methods). Each point denotes the fraction colocalized per image, n. Here, $n = 21$ and 23 for WT and $CLN3^{+}/BNI1^{+}$, respectively. For the boxplots, the median is shown as a colored horizontal line, whiskers show the 1.5 interquartile range, and the bottom and top of each box are the 25th and 75th percentiles, respectively. Any points beyond the whiskers are outliers. Source data are provided as a Source Data file.

Supplementary References

1 Choi, J.-M., Dar, F. & Pappu, R. V. LASSI: A lattice model for simulating phase transitions of multivalent proteins. *PLOS Computational Biology* **15**, e1007028, doi:10.1371/journal.pcbi.1007028 (2019).

2 Kar, M. *et al.* Phase separating RNA binding proteins form heterogeneous distributions of clusters in subsaturated solutions. *Proceedings of the National Academy of Sciences* **119**, e2202222119, doi:https://doi.org/10.1073/pnas.2202222119 (2022).

3 Lu, T. & Spruijt, E. Multiphase Complex Coacervate Droplets. *Journal of the American Chemical Society* **142**, 2905-2914, doi:10.1021/jacs.9b11468 (2020).

4 Gerlach, W. & Giegerich, R. GUUGle: a utility for fast exact matching under RNA complementary rules including G–U base pairing. *Bioinformatics* **22**, 762-764, doi:10.1093/bioinformatics/btk041 (2006).

5 Deigan, K. E., Li, T. W., Mathews, D. H. & Weeks, K. M. Accurate SHAPE-directed RNA structure determination. *Proceedings of the National Academy of Sciences* **106**, 97-102, doi:doi:10.1073/pnas.0806929106 (2009).

6 Smola, M. J., Rice, G. M., Busan, S., Siegfried, N. A. & Weeks, K. M. Selective 2′-hydroxyl acylation analyzed by primer extension and mutational profiling (SHAPE-MaP) for direct, versatile and accurate RNA structure analysis. *Nature Protocols* **10**, 1643-1669, doi:10.1038/nprot.2015.103 (2015).

7 Langdon, E. M. *et al.* mRNA structure determines specificity of a polyQ-driven phase separation. *Science* **360**, 922-927, doi:10.1126/science.aar7432 (2018).

8 Boeynaems, S. *et al.* Spontaneous driving forces give rise to protein-RNA condensates with coexisting phases and complex material properties. *Proceedings of the National Academy of Sciences USA* **116**, 7889-7898, doi:doi:10.1073/pnas.1821038116 (2019).
