# Supplementary material for: Dynamical control enables the formation of demixed biomolecular condensates
Source: Nat Commun. 2023 Nov 24;14:7678. doi: 10.1038/s41467-023-43489-4 (PMC10667521; doi:10.1038/s41467-023-43489-4)
Supplement: Supplementary file 3 — Reporting Summary [file 41467_2023_43489_MOESM3_ESM.pdf]

nature portfolio

Corresponding author(s): Rohit Pappu
Last updated by author(s): Oct 25, 2023

Reporting Summary

Nature Portfolio wishes to improve the reproducibility of the work that we publish. This form provides structure for consistency and transparency in reporting. For further information on Nature Portfolio policies, see our Editorial Policies and the Editorial Policy Checklist.

Statistics

For all statistical analyses, confirm that the following items are present in the figure legend, table legend, main text, or Methods section.

n/a	Confirmed	
☐	☒	The exact sample size (*n*) for each experimental group/condition, given as a discrete number and unit of measurement
☐	☒	A statement on whether measurements were taken from distinct samples or whether the same sample was measured repeatedly
☐	☒	The statistical test(s) used AND whether they are one- or two-sided<br>*Only common tests should be described solely by name; describe more complex techniques in the Methods section.*
☒	☐	A description of all covariates tested
☒	☐	A description of any assumptions or corrections, such as tests of normality and adjustment for multiple comparisons
☐	☒	A full description of the statistical parameters including central tendency (e.g. means) or other basic estimates (e.g. regression coefficient) AND variation (e.g. standard deviation) or associated estimates of uncertainty (e.g. confidence intervals)
☐	☒	For null hypothesis testing, the test statistic (e.g. *F*, *t*, *r*) with confidence intervals, effect sizes, degrees of freedom and *P* value noted<br>*Give P values as exact values whenever suitable.*
☒	☐	For Bayesian analysis, information on the choice of priors and Markov chain Monte Carlo settings
☒	☐	For hierarchical and complex designs, identification of the appropriate level for tests and full reporting of outcomes
☐	☒	Estimates of effect sizes (e.g. Cohen's *d*, Pearson's *r*), indicating how they were calculated

Our web collection on statistics for biologists contains articles on many of the points above.

Software and code

Policy information about availability of computer code

Data collection	We used the Region of Interest (ROI) and Time Analysis tools within the Nikon Imagine Software suite to trace fluorescence intensity of photobleached spots, unbleached condensates, and a region with no condensate. We used the Big-FISH Python package to identify CLN3 and BNI1 spots within Ashbya cells. BigFISH implements a spot detection algorithm for smFISH that identifies puncta locations. Nuclei detection was performed using the Cellpose 2.0 Python package.
Data analysis	Matlab was used to perform the pixel shift and proximity analysis from the extracted puncta locations. The corresponding images were generated using Matplotlib and Matlab. Adobe Illustrator was used for editing legend locations and labels. The GUUGle web server was used to identify complemtary sites between pairs of RNAs. We used LaSSI, an open-source lattice simulation engine to simulate ternary systems and invetigate minimal models of associative polymers that lead to equilibrium demixed condensates (https://github.com/Pappulab/LASSI). We used the Numpy, Matplotlib, and Seaborn Python packages as well as Adobe Illustrator to create the respective figures. We used FIJI and the Coloc2 plugin in FIJI to analyze phase separation and colocalization data respectively. All code needed to reproduce figures in this manuscript have been deposited at https://github.com/Pappulab/dynamical-control and is publicly available as of the date of publication.

For manuscripts utilizing custom algorithms or software that are central to the research but not yet described in published literature, software must be made available to editors and reviewers. We strongly encourage code deposition in a community repository (e.g. GitHub). See the Nature Portfolio guidelines for submitting code & software for further information.

Data

Policy information about availability of data

All manuscripts must include a data availability statement. This statement should provide the following information, where applicable:

- Accession codes, unique identifiers, or web links for publicly available datasets
- A description of any restrictions on data availability
- For clinical datasets or third party data, please ensure that the statement adheres to our policy

Raw experimental images, phase separation data, fluorescence recovery after photobleaching, and additional data needed to reproduce figures in this manuscript have been deposited in the Source Data file and / or at https://github.com/Pappulab/dynamical-control, which is publicly available as of the date of publication and has been archived in Zenodo with the identifier https://doi.org/10.5281/zenodo.1004269. Protein and RNA expression constructs for in vitro measurements are available upon request. Correspondence and requests for materials should be addressed to A.S.G. and R.V.P.

Human research participants

Policy information about studies involving human research participants and Sex and Gender in Research.

Reporting on sex and gender	N/A
Population characteristics	N/A
Recruitment	N/A
Ethics oversight	N/A

Note that full information on the approval of the study protocol must also be provided in the manuscript.

Field-specific reporting

Please select the one below that is the best fit for your research. If you are not sure, read the appropriate sections before making your selection.

[x] Life sciences [ ] Behavioural & social sciences [ ] Ecological, evolutionary & environmental sciences

For a reference copy of the document with all sections, see nature.com/documents/nr-reporting-summary-flat.pdf

Life sciences study design

All studies must disclose on these points even when the disclosure is negative.

Sample size	A minimum of three biological replicates, each with a minimum of three technical replicates were taken for each sample. These sample sizes appear sufficient given the variances observed across replicas.
Data exclusions	Images with uneven illumination and few regions of non- overlapping hyphae were discarded. Images in which the number of CLN3 and BNI1 spots were outside an order of magnitude of each other were excluded.
Replication	A minimum of three technical replicates were taken for each sample and standard error of means are shown. All attempts at replication were successful.
Randomization	Allocation was not random as all experimental groups in this study were clearly defined by the constructs / strains used in each experiment.
Blinding	Blinding was not relevant for this study because the outcomes of each experiment could be measured objectively and each sample was prepared and measured in an identical manner.

Reporting for specific materials, systems and methods

We require information from authors about some types of materials, experimental systems and methods used in many studies. Here, indicate whether each material, system or method listed is relevant to your study. If you are not sure if a list item applies to your research, read the appropriate section before selecting a response.

Materials & experimental systems

n/a	Involved in the study	
☒	☐	Antibodies
☒	☐	Eukaryotic cell lines
☒	☐	Palaeontology and archaeology
☒	☐	Animals and other organisms
☒	☐	Clinical data
☒	☐	Dual use research of concern

Methods

n/a	Involved in the study	
☒	☐	ChIP-seq
☒	☐	Flow cytometry
☒	☐	MRI-based neuroimaging
